# Assessment of productivity of *Culex* spp. larvae (Diptera: Culicidae) in urban storm water catch basin system in Wrocław (SW Poland)

**DOI:** 10.1007/s00436-016-4912-x

**Published:** 2016-01-25

**Authors:** Katarzyna Rydzanicz, Piotr Jawień, Elżbieta Lonc, Magdalena Modelska

**Affiliations:** Department of Microbial Ecology and Environmental Protection, Institute of Genetics and Microbiology, University of Wrocław, 63/77 Przybyszewskiego Street, 51-148 Wrocław, Poland; Institute of Geological Sciences, Environmental Geology Laboratory, University of Wrocław, Max Born Square 9, 50-205 Wrocław, Poland

**Keywords:** *Culex pipiens* s.l, *Cx. torrentium*, Mosquitoes in Poland, Catch basins, Seasonal and spatial distribution of larvae

## Abstract

In urban environments, catch basins serve as major developmental and resting sites for anthropophilic and zoophilic mosquitoes. However, the use of this habitat is inconsistent, with abundance of larvae varying significantly across catch basins at a fine spatial scale. During seasonal summer investigations on mosquito species composition, their spatial and temporal distribution and the environmental characteristic of the breeding sites in the underground storm drain systems of the Wrocław urban area (SW Poland) were assessed from May to September in 2012–2013. The study was conducted in order to develop a rational strategy to control mosquito populations and prevent the potential human exposure to mosquito-transmitted pathogens. Mosquito larvae and pupae were collected and identified weekly from 100 regularly inspected street catch basins located in the town center. All existing and potential breeding habitats in the study area were recorded using a GPS receiver (Magellan MobileMapper CX) and transferred to the computer database. Collected data on the geographical location of inspected breeding places, water quality parameters in inspected catch basins, daily temperature, and precipitation were imposed on orthophotomap in ArcGIS (ESRI, USA). Water quality parameters including pH, electrical conductivity, and water temperature were measured by standard methods. Chemical water analysis of cations (Na^+^, NH_4_^+^, K^+^, Mg^2+^, Ca^2+^) and anions (Cl^−^, NO_2_^−^, NO_3_^−^, SO_4_^2−^) were carried out using Waters Alliance high-performance liquid chromatograph (HPLC) 2695 with 432 Conductivity Detector and 2998 Photodiode Array Detector, an IC-Pak Anion HR column (glauconate/borate eluent) and IC-Pak Cation M/D column (EDTA/HNO_3_ eluent). Over two seasonal studies and 3739 samplings in total, 3669 mosquito larvae and 274 pupae/1 dip (from 0 to 110 individuals/dip) were collected by dipper. *Culex pipiens* s.l. (L.) and *Cx. torrentium* (Martini) prevailed at all catch basins of the study area as the predominant species. In all examined catch basins, autogenous individuals dominated by far. Breeding activity was first detected in early May. Peak abundance of *Culex* spp. population in many catch basins was observed in June 2012 and August 2013 when average daily temperatures were increasing and rainfall had declined. Dry periods between rainfalls varied during 2 years of the study period and were noted on June 2012 as well as on July and August 2013. Organically enriched catch basins with significant higher concentrations of Na^+^ and NO_3_^−^ were found to be more productive breeding habitats. Differences in the *Culex* immature stage density based on the variables of habitat type, temperature, and precipitation support the need for ongoing surveillance in communities to guide public health officials in planning for and prioritizing mosquito control efforts.

## Introduction

It is known that mosquitoes serve as vectors for a wide variety of human and veterinary pathogens that range from the parasites causing malaria and filariasis to those responsible for hemorrhagic and many encephalitic viral infections. Scientists and the public are concerned on the increasing risk of mosquito vector-borne diseases in Europe, including central Europe (Becker 2009; ECDC 2007, 2012). Changing climate conditions make diseases, which have already been eradicated, or newly appearing diseases a threat to human health. It is necessary to inform the public of the potential risk of introduced or newly occurring infection diseases or vector organisms on the basis of realistic assessments in order to prevent a sensitive public from overreacting, due to panic mongering.

According to Norris (2004), both climate and land use changes have an enormous impact on local ecology and habitats that affect mosquito abundance, species composition, and, ultimately, pathogen transmission. Historic examples of these modifications include impoundments, dams, and irrigation systems that create havens for the mosquitoes that transmit malaria, dengue, and filariasis. One of the environmental modifications that is somewhat unique to urban settings worldwide are the kilometers of pipe underneath the streets of almost every large city that constitute the subterranean storm water handling system including catch basins and manhole chambers, all of which drain runoff water from residential, business, and commercial establishments as well as highways and streets. These massive systems collect organic material together with the surface water from precipitation, street wash, car wash, lawn irrigation, and other sources (Su et al. 2003). Despite the elaborate design to enhance the drainage efficiency, some features such as minimum grade, accumulation of debris and trash, and irregular water flow result in puddling and impoundment of drained water both in the underground and aboveground structures.

The underground parts of the mentioned drainage systems with stable microclimatic conditions serve as major developmental and resting sites for many anthropophilic and zoophilic mosquitoes (Becker et al. 2012). They are notorious vectors of many viruses such as Ross River virus in Australia, Sindbis virus (SINV) in the Old World, West Nile virus (WNV) in both the Old and New World, and Usutu virus in Europe (Pfunter 1978; Chanda and Shisler 1980; Mulligan and Schaefer 1981; Smith and Shisler 1981; Byrne and Nichols 1999; Kurkela et al. 2008; Jöst et al. 2011; Vázquez et al. 2011). Mosquito breeding and/or resting sites in massive storm drain systems are especially difficult to control in terms of accessibility, irregular timing of flooding, and fluctuation of water amount and thus cause considerable public health concerns from the standpoint of arbovirus transmission (Mulligan and Schaefer 1982; Marfin et al. 1993; Kronenwetter-Koepel et al. 2005). Environmental features may influence viral risk of human illness by altering the transmission competence and fitness of vectors, the infection prevalence in the vertebrate reservoir host population, and the abundance and spatial distribution of all three organisms involved in the arboviral transmission cycle: hosts, parasites, and vectors. To provide a basis for the management control of mosquitoes in these vast systems, detailed biological and ecological surveys are needed.

Wrocław city (ca. 640,000 inhabitants), the capital of Lower Silesia is part of Plain Wrocław (318.53), in the Silesian Lowland (318.5), located at south-western Poland has an elevation of 120–150 m above sea level and is divided into two unequal parts by the wide Odra River valley (Kondracki 2011; Weitzel et al. 2015). It is located in a transitional climate zone with the conditions changing from more maritime in the west to more continental in the east (Kiewra et al. 2014). This results in more severe winter conditions in the east and higher rainfall in the west across Lower Silesia. The annual mean air temperature in the lowland area of the region exceeds 8 °C. The coldest month January (monthly average air temperature below 0 °C) and the warmest July (monthly average air temperature close to 20 °C) with the annual average rainfall between 500 and 580 mm in the lowland area of Lower Silesia support seasonal mosquito development.

The most common aquatic habitats in the Wroclaw region are the floodplains and the Rivers Odra, Sleza, Bystrzyca, Widawa, and Olawa (Rydzanicz and Lonc 2003). These floodplains include an extensive system of ditches and tributaries in and around the city of Wroclaw. These water bodies represent hundreds of permanent microsystems and macrosystems (ponds, marshes, potholes, pools, wells, and drainage canals) that are suitable habitats for mosquito breeding. The largest and most important mosquito breeding habitats are located in the irrigation fields, situated in the north-western part of Wroclaw (Rydzanicz et al. 2009).

The aim of our study was to determine the spatial and temporal distribution of mosquitoes in the underground storm drain systems of the Wrocław urban area (SW Poland), including species composition and population dynamics of immature stages, as well as to assess an impact of some abiotic factors conducive to underground mosquito production.

## Materials and methods

### Sampling of immature mosquitoes and species identification

**Fig. 1:**
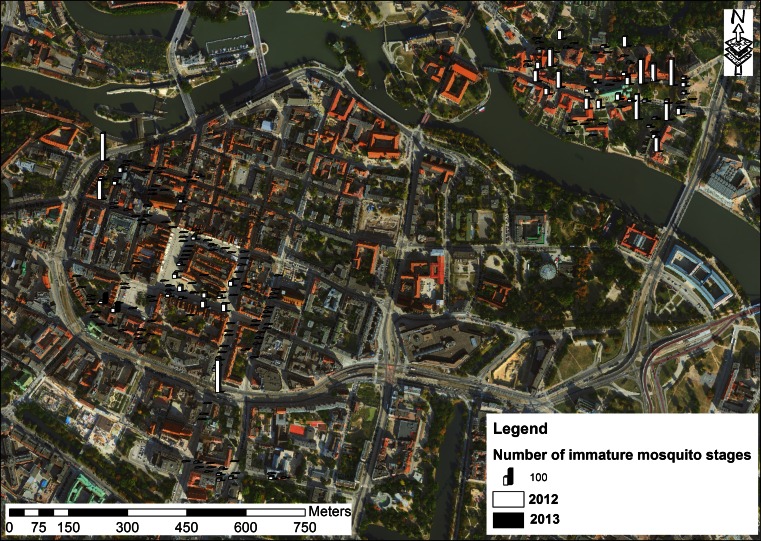
Map of Wroclaw, where all catch basins were located in the city center. The number of immature mosquito stages as well as the number of breeding places with larval development varied during two seasons of the study period

The density of mosquito developmental stages and their breeding site preferences were investigated twice a month in the relatively uniform 100 city storm water catch basins (average chamber size 0.4 × 0.6 m with grill cover) from 1 May to 30 September 2012 and 2013. In total, 3739 inspections during 21 weeks were made. All catch basins were located in the city center of Wrocław (Market Square and the Cathedral Island) that is important from the business and tourist points of view (Fig. 1). Larvae and pupae were collected using a 350-ml mosquito dipper.

All existing and potential breeding habitats in the study area were recorded using a GPS receiver (Magellan MobileMapper CX) and transferred to the computer database. Collected data on the geographical location were imposed on orthophotomap in ArcGIS (ESRI, USA). The database was constructed in the tabulated form with collected data during field visits, including collection date, catch basin location, name of the street, number of immature mosquito stages in each of the visited manholes, and value of the water quality parameters. In the study period (from May to September 2012–2013), meteorological data on average daily precipitation and temperature in Wrocław area were obtained from the Department and Observatory of Climatology and Meteorology of the Wrocław University.

Collected *Culex* mosquito specimens, including early (1st and 2nd) and late (3rd and 4th) instars, as well as pupae were counted and tested for autogeny according to Becker et al. (2012). After rearing the juvenile stages to adults, the populations were examined in regard to stenogramy and autogeny. A population was assessed as anautogenous when no egg rafts occurred at all, even 4 weeks after the emergence of the latest adults.

A subsample of randomly selected fourth instar larvae were determined according to Becker et al. (2010) by the number of setae 1 at the abdominal segments III to V. *Culex pipiens* s.l. larvae possess regularly two or two and three setae at each place, whereas *Culex torrentium* is described to have each four setae at each place. A total of 17 larvae collected from catch basins in the Market Square area were identified to species by enzyme electrophoresis (Weitzel et al. 2015).

### The analysis of water quality parameters

Water quality parameters including pH, electrical conductivity, and water temperature were measured during the field study by Hanna Combo pH and EC System (HI 98129, USA). Chemical water analysis of cations (Na^+^, NH_4_^+^, K^+^, Mg^2+^, Ca^2+^) and anions (Cl^−^, NO_2_^−^, NO_3_^−^, SO_4_^2−^) were carried out in the Environmental Geology Lab of the Institute of Geological Sciences of Wrocław University, using Waters Alliance high-performance liquid chromatograph (HPLC) 2695 with 432 Conductivity Detector and 2998 Photodiode Array Detector, an IC-Pak Anion HR column (glauconate/borate eluent), and IC-Pak Cation M/D column (EDTA/HNO_3_ eluent). The detailed analysis of ions in wastewater was carried out for a selection of 28 catch basins that were most preferable and non-preferable for mosquito immature stage development during the study period in 2012–2013. Before measurements, each water sample was filtered through a syringe filter (diameter of pores 0.45 μm). The analytical precision of these ions was 0.01 mg dm^−3^. Using a 100-μL injection, the estimated method’s detection limits (MDLs), as parts per billion, were as follows: Cl^−^ 50, NO_2_^−^ 50, NO_3_^−^ 75, SO_4_^2−^ 75, K^+^ 15, Na^+^ 5, Mg^2+^ 10, NH4^+^ 5, and Ca^2+^ 15. Concentrations of HCO_3_^−^ were measured as total alkalinity. A water sample of 100 ml was used for titration using 0.1 M HCl in the presence of methyl orange. The alkalinity, in meq/L units, was calculated using the gram equivalent of HCO_3_^−^. The analytical precision was better than 3 mg/L.

Differences between ion concentrations in wastewater collected from 28 most preferred and non-preferred catch basins were analysed using *U* Mann–Whitney test. Differences were considered significant if *p* < 0.05. Statistical analyses were computed using the Statistica 7.0 software package.

## Results

In total, we collected 3669 mosquito larvae and 274 pupae from 3739 inspected catch basins in the study area of Wrocław. The sample size varied between 0 and 110 individuals per dip. However, the number of immature mosquito stages as well as the number of breeding places with larval development varied during two seasons of the study period 2012–2013 (Fig. 1, Table 1). The density of immature stage population was higher in 2012 (2.1 specimens/dip) despite lower average values of daily temperature and precipitation, reaching 16.5 °C and 2.2 mm in 2012, while in 2013 daily temperature and precipitation were 18.2 °C and 3.2 mm, respectively.

**Table 1:** Comparison of the number of catch basins positive for *Culex* spp. developmental stages and environmental conditions influencing the mosquito development in Wrocław area during the study period (May–September 2012–2013)

Year of the study	Average daily		Number of catch basins positive for immature mosquito stages development	Average number (%) of mosquito larvae and pupae/dip
	Precipitation [mm]	Temperature [°C]		
2012	2.2	16.5	103	2.1 (84.0)
2013	3.2	18.2	310	0.4 (16.0)
Average/total	2.7/–	17.3/–	–/413	–/2.5

All larvae and pupae collected from 413 positive catch basins were *Cx. pipiens* s.l./*Cx. torrentium*. During two seasonal study periods (2012–2013), no immature stages of other dipterans were found at the randomly distributed catch basins (Fig. 1).

In all examined catch basins, autogenous individuals dominated by far. Depending on the location, the percentage of autogenous mosquitoes from populations in the underground system varied from 0 to 80 %.

**Fig. 2:**
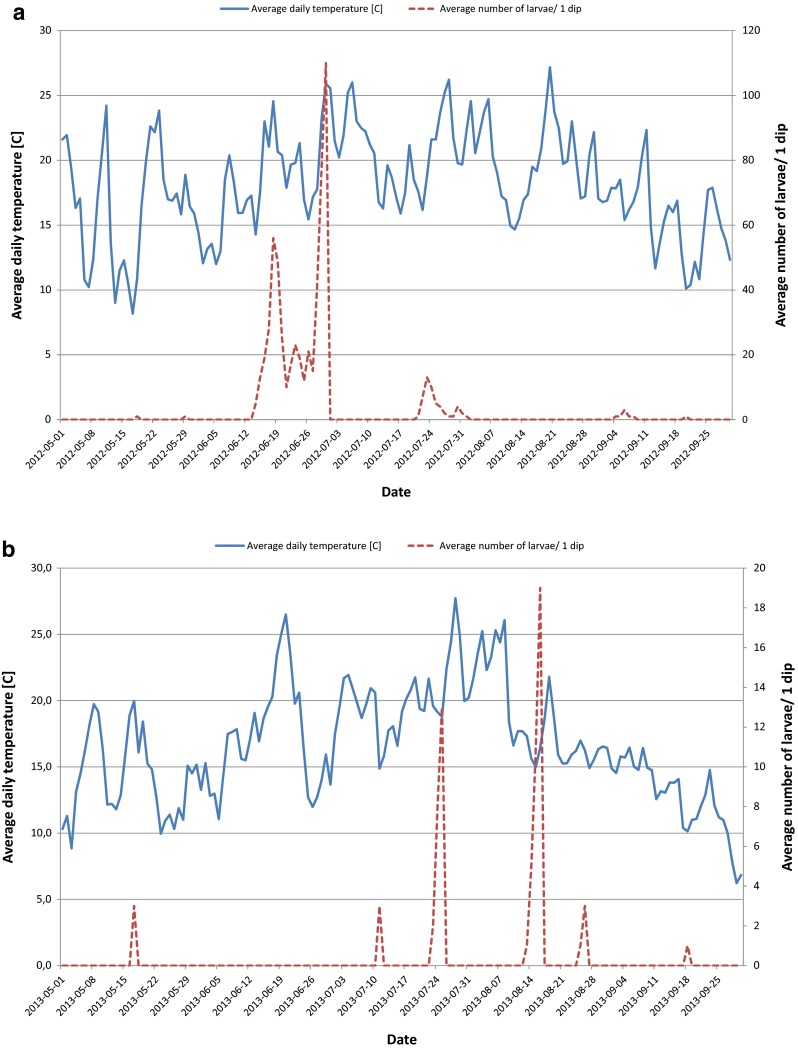
**a** Relationship between average daily temperatures and average numbers of mosquito larvae/1 dip from May to September 2012. **b** Relationship between average daily temperatures and average numbers of mosquito larvae/1 dip from May to September 2013

No immature mosquito stage breeding in catch basins were found until the second week of May 2012 and 2013 (Fig. 2a, b).

**Fig. 3:**
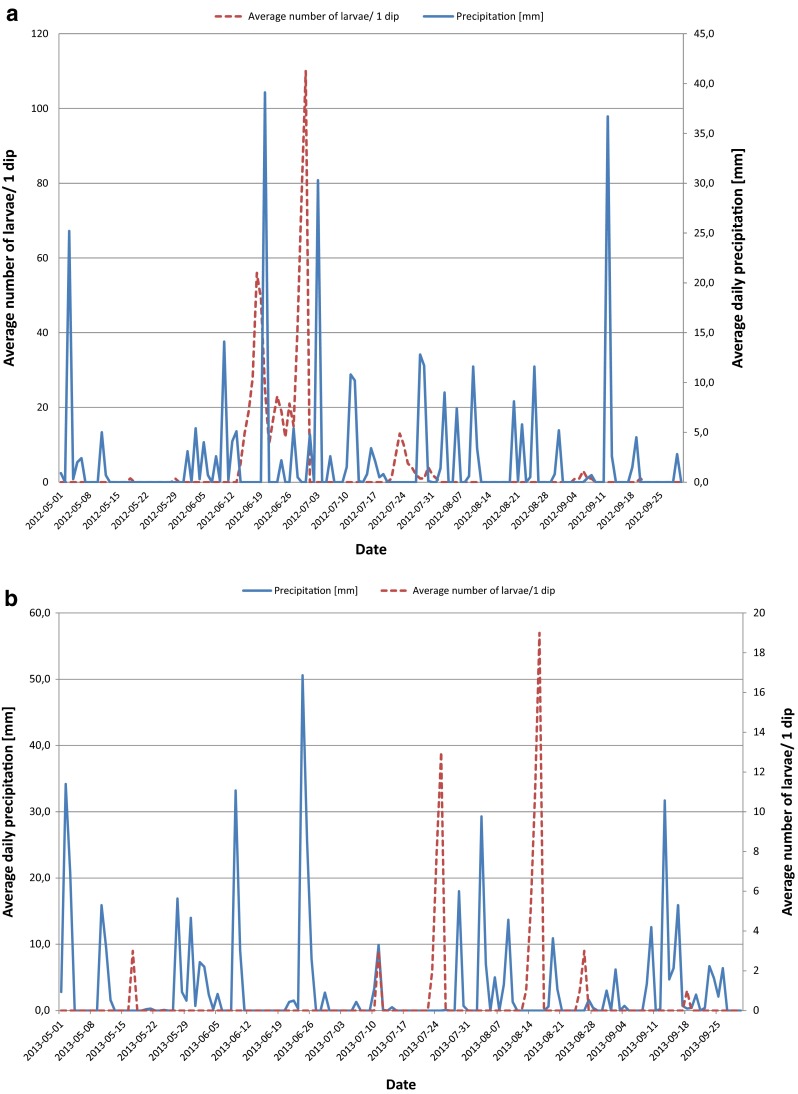
**a** Relationship between average daily precipitation [mm] and average numbers of mosquito larvae/1 dip from May to September 2012. **b** Relationship between average daily precipitation [mm] and average numbers of mosquito larvae/1 dip from May to September 2013

The initial peak of mosquito developmental stages was observed on 18 and 29 May 2012 and on 17 May 2013 when the average daily temperature reached 10.9, 18.9, and 20 °C, respectively. Mosquito productivity in many catch basins increased during the dry periods between rainfalls. The *Culex* spp. population peaked significantly at 56 larvae/dip and 110 larvae/dip in 18 and 30 June 2012 when the average daily temperatures were 25.6 and 26 °C (Fig. 3a, b).

However, in 2013, the density of immature stage population was the highest in July and August with two peaks of abundance up to 13 larvae/dip to 19 larvae/dip. At that time, immature stage development was followed by average daily temperature ranging from 16.5 to 18.8 °C (Fig. 3a, b).

The most extensive rainfall period was observed on 20 June 2012 and 3 July 2012 (39.1 and 30.3 mm) as well as on 9 and 24 June 2013 (3.2 and 50.6 mm), causing a reduction of larval development or “flushing” mosquito larvae from underground drain system (Fig. 3a, b).

During the study period, mosquito larvae were found in catch basins characterized by a wide range of water temperatures (from 9.8 to 23.5 °C), corrected electrical conductivity (from 59 to 4000 μS), and pH value (from 5.0 to 9.7). The catch basin sites that supported mosquito larvae development during the study period contained a higher average amount of cations (Na^+^, Mg^2+^, Ca^2+^) and anions (Cl^−^, NO_2_^−^, SO_4_^2−^) as well as electrical conductivity (Table 2). However, both catch basin categories differed significantly (*p* < 0.05) regarding Na^+^ and NO_3_^−^concentration.

**Table 2:** Average value of water parameters in catch basin sites preferable and non-preferable by immature mosquito stages during the study period (2012–2013)

Catch basin type	Water parameter										
	Cations [mg/dm^3^]					Anions [mg/dm^3^]				pH	Electrical conductivity [μS]
	Na^+^	K^+^	Mg^2+^	Ca^2+^	NH4^+^	Cl^−^	NO_2_^−^	NO_3_^−^	SO_4_^2−^		
Productive	71.85	5.06	1.32	26.01	6.44	43.64	0.81	0.86	12.78	8.27	318.78
Non-productive	30.23	6.0	1.21	23.62	7.08	26.11	0.46	2.4	8.72	8.31	226.07
Differences in ion concentration	−1.99*	0.80	−0.16	−0.21	−0.24	−1.22	−0.87	−2.13*	−1.77	–	–

*p < 0.05

It was also observed that those breeding places were often organically enriched by food scraps (data not presented).

## Conclusions

The contribution of subterranean and surface storm water systems to vector abundance and potential disease transmission is known only in some regions (Norris 2004). It is well documented that catch basins are convenient sites for the development of the larvae of many mosquito species, both indigenous and invasive in the USA, Europe, and Japan (Anderson et al. 2006; Bellini et al. 2009; Kobayashi et al. 2008; Andreadis and Wolfe 2010; Becker et al. 2012). Probably, the introduction to many European countries with exotic mosquito species such as *Aedes albopictus* (Skuse) and *Ochlerotatus japonicus japonicus* (Theobald) is affected by many different factors such as the transformation of environment, climate change, and globalization (Scholte et al. 2007; Pluskota et al. 2008; Becker 2009). Due to outstanding adaptability, these species colonize new ecological niches, creating previously unknown risks to human health because of pathogen transmission, including several arboviruses such as dengue, West Nile, and Chikungunya (Medlock et al. 2012; Schaffner et al. 2013).

The abundance of the *Culex* specimens in Wrocław confirms that storm water catch basins provide suitable but unstable conditions for mosquito productivity. Additionally, in our study, mosquito larva populations were not uniformly distributed, both temporarily and spatially. According to Kronenwetter-Koepel et al. (2005), there are several possible explanations for these observations. The authors assume that catch basins in high-intensity urban areas contain less organic debris (e.g., leaves, grass clippings) as well as less areas that contain vegetation for mosquitoes to rest during the hottest, driest times of the day. Thus, adult female mosquitoes have less available blood meal sources (e.g. lower abundance of birds and small mammals). Finally, catch basins in high-intensity urban areas may have a higher volume of water flowing into them because there is more impervious surface area and less lawn and other green space to absorb water resulting in more rigorous flushing of the basins during rainfall.

In our study, the majority of sampled larvae were *Cx. pipiens* s.l.*/Cx. torrentium*; however, larvae identified as *Cx. torrentium* were only occasionally found (Weitzel et al. 2015). Further study on the proportion between both species density within the catch basis system in Wrocław area will be carried out. It is known that *Cx. torrentium,* a morphologically very similar and cryptic species, has ability for enzootic transmission of the SINV (Ockelbo disease). In northern Europe, it caused regular outbreaks in humans every 7 years (Kurkela et al. 2008; Reusken et al. 2011).

According to Weizel et al. (2011) and Hesson et al. (2014), both species occur together in large areas of Europe, and *Cx. torrentium* dominates in northern Europe and *Cx. pipiens* dominates south of the Alps. The transition in dominance occurs in central Europe, where both species are roughly equally common. The authors suggest a strong correlation between the length of the growing season at different sites and occurrences of the two species. As the growing season increases, the proportion and detection of *Cx. torrentium* decrease, whereas those of *Cx. pipiens* increase. These findings have important consequences for the interpretation of the results of studies on major enzootic and bridge vectors of mosquito-borne bird-associated viruses (i.e., Sindbis, West Nile, and Usutu viruses), especially in central Europe and Scandinavia.

There has been a great deal of concern around WNV and its potential spread in Europe. *Culex pipiens* has been implicated in its transmission ecology, whereas the potential role of *Cx. torrentium* has been largely ignored (Lundström et al. 2013). The epidemic role of *Cx. pipiens pipiens* was recorded since XX century in the USA (Geery and Holub 1989; Varuni et al. 2001; Anderson et al. 2011; CDC 2011) as well as in some European countries (Hubálek and Halouzka 1999), like Italy (1998), Czech Republic (1997), Romania (1996), Slovakia (1970–1973), and Russia in the Volga Valley region (1963–1968). Also, data from Greece and Serbia confirm high WNV infection rate in *Cx. pipiens* and *Anopheles maculipennis* s.l. females that may result in explosive spread of WNV and be an alarming signal for neighboring countries (Chaskopoulou et al. 2011; Kamenesi et al. 2014).

In the present Wrocław study, it has been shown that larvae and pupae of *Cx. pipiens* s.l.*/Cx. torrentium* were present from May to September with extensive population peaks from June to August 2012 and 2013. This seasonal pattern of developmental stages of *Cx. pipiens* s.l.*/Cx. torrentium* in Wrocław was similar to the gonotrophic activity of *Cx. quinquefasciatus* (Say) observed by Strickman (1988) in San Antonio (Texas) and by Su et al. (2003) in Orange County (southern California). The authors observed active female oviposition and reproduction at higher temperatures (30 °C) during summer months while mosquito larvae and pupae were present from April to October, with peaking during May–September.

During the study period (2012–2013) in Wroclaw, most of the samples showed low larval counts ranging from 0 to 110 larvae and pupae/dip (per average from 0.4 to 2.1 immature stages/dip). However, considering the massive occurrence in underground storm drain systems, these low larval counts can aggregate to high numbers in the area as it was also observed by Su et al. (2003).

According to Gardner et al. (2012), weather is one of the most important predictors of *Culex* larval abundance in the storm water catch basin system, explaining much of the temporal variation in urban larval production. Large, multi-hour rainfall events within 4 days led to “flush” almost all mosquito larvae from underground storm drains and to limit adult production in catch basins for up to a week in metropolitan Chicago. High ambient and aquatic temperatures have also been shown to accelerate larval production and development rates. These effects do not influence all catch basins equally: some catch basins are protected from heat and rainfall by overhanging trees, and catch basins surrounded by impervious surfaces are more susceptible to street chemical and fertilizer runoff due to high rainfall than those surrounded by grass.

Our study demonstrates also that *Cx. pipiens* s.l.*/Cx. torrentium* larvae were tolerant to a wide range of water quality parameters in catch basin sites, i.e. ion concentration, pH, and electrical conductivity. It can be related to the physical and hydrological characteristics of these aquatic systems, like low air flow, water stagnation, low evaporation, and high pollution. The underground storm drain systems of Wrocław were partly inhabited by autogenous *Cx. pipiens* s.l. populations producing stenogamous females which do not hibernate in state of diapauses and bite humans. According to Becker et al. (1999), autogeny is genetically determined. In underground breeding sites, vertebrates for a blood meal do not occur or are rare with the exception of the occurrence of rats. Other environmental factors such as larval nutrition, isolation of the breeding site, or photoperiod influence larval development. In these highly eutrophic breeding sites, larvae are able to develop a prominent fat body which allows females of *Cx. pipiens* biotype *molestus* to lay the first egg batch without blood meal (Becker et al. 2012).

In contrast, *Cx. torrentium* is described as a “wild mosquito,” which is not as synanthrope as *Cx. pipiens* s.l. Several studies identified a wide variety of breeding sites for both species including artificial habitats (Gillies and Gubbins 1982; Hesson et al. 2011; Lühken et al. 2015; Weizel et al. 2011). Hesson et al. (2014) and Weitzel et al. (2015) found *Cx. torrentium* to be even more frequent in artificial compared to natural breeding sites. However, Lühken et al. (2015) did not find differences regarding the breeding sites of *Cx. pipiens* s.l. and *Cx. torrentium*, which were both present in different water bodies in Germany, irrespective of the span measured for most of the ecological parameters such as size of the water body, water depth, percentage cover of plants, pH, conductivity and salinity, and temperature and oxygen content.

On the other hand, Gardner et al. (2013) mention the important positive predictors of high larval abundance in catch basins in temperate climate zone (Chicago, IL) like aquatic ammonia and nitrates and occurrence of shrubs of height <1 m surrounding the catch basins. Also, in the study carried out by Geery and Holub (1989) in western Cook County, IL, on determining seasonal larval production in regularly examined street catch basins, it showed that the abundance of *Cx. pipiens* and *Cx. restuans* (Theobald) larvae within catch basins was not correlated with water pH and only showed a weak, positive correlation with water temperature. Nevertheless, it is noteworthy that the important changes in water quality parameters can be caused by the rain dilution when water is collected from above ground and drained into underground systems, washing out debris and organic matter (Su et al. 2003).

According to Kronenwetter-Koepel et al. (2005), the comprehensive mosquito surveillance including catch basin study provides basis for rational mosquito prevention and control strategies by public health and government officials. Surveillance has become increasingly important since the introduction of WNV into North America in 1999. Application of effective insecticides in a timely manner to catch basins in cities can reduce the number of adult mosquitoes and reduce risk of the transmission of WNV in urban areas (Anderson et al. 2011).

Biological control of mosquito larvae within the underground storm sewer system is based on using different formulations (liquids, tablets, granules) of both microbial insecticide containing *Bacillus thuringiensis israelensis* and *Bacillus sphaericus* (eg., VectoBac 12AS, VectoBac CG, VectoLex CG, VectoLex WSP, VectoMax WSP), neurotoxins obtained by fermentation of *Saccharopolyspora spinosa* (Spinosad), insect growth regulators such as methoprene or diflurobenzuron (Hazelrigg and Pelsue 1980; Su 2008; Anderson et al. 2011). It is noticeable that the regular application of these larvicides can reduce larval development in the period from 1 to 6 weeks (Su 2008; Bellini et al. 2009; Anderson et al. 2011). It is also considered that the persistence of organic deposits in the wells (such as leaves or food residue) can support and prolong the action of certain mixtures of active substances, mainly of insect growth regulators (Backer and Yan 2010).

The environmental factors, such as the frequency and intensity of rainfall, high and average daily ambient temperature, and the size of the doses, may on the one hand support the rapid larval development of various mosquito species within the underground drainage system and on the other hand determine the effectiveness of these insecticides (Koenraadt and Harrington 2008; Gardner et al. 2012). The timing and amount of rainfall is a continuous challenge to those in charge of mosquito control programs. It was also confirmed in Stratford CT by Anderson et al. (2011). According to Su et al. (2003) rainfall van affect numbers of juvenil mosquitoes in catch basins and storm drains in three ways: (1) flushing larvae into locations unfavorable for survival, (2) draining toxic chemicals from road areas into catch basins and drains, and (3) changing the water quality that may enhance or hinder growth of mosquitoes. Additionally, water is needed in the catch basins to ensure that mosquitoes breed continuously and reach adulthood.

Therefore, reliable surveillance data on the distribution of mosquito larvae in manhole chambers with special regard to environmental factors, such as irregular timing of flooding, are necessary to provide straightforward implementation of control operations against mosquito larvae and preventing potential epidemiological risk of mosquito-borne diseases in urban areas.

**Fig. 4:**
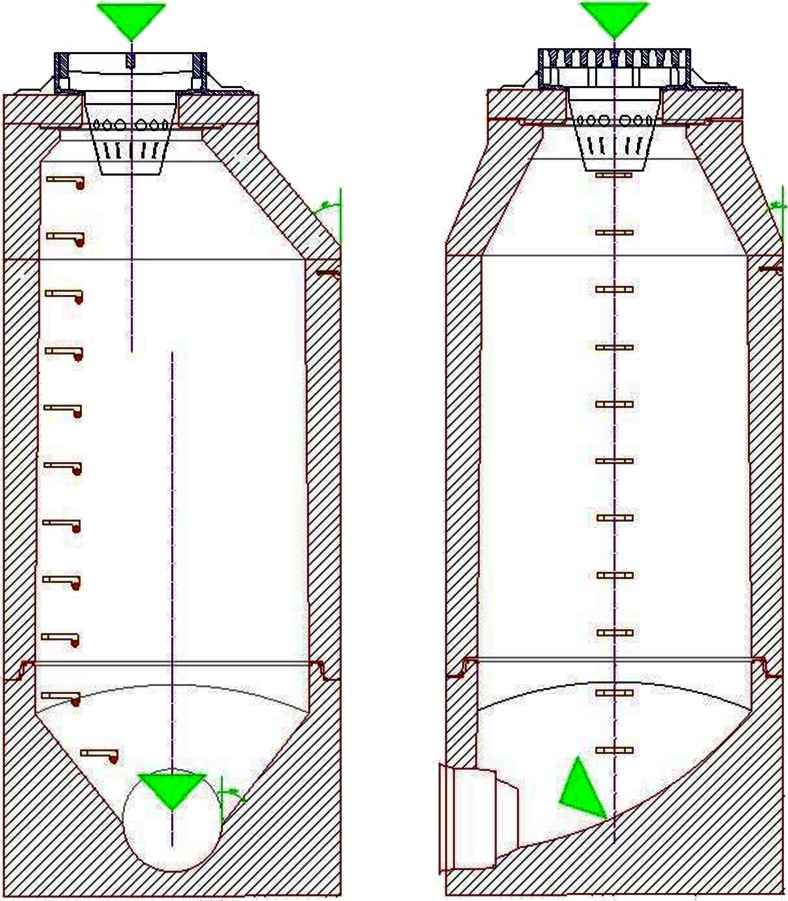
The catch basin model preventing mosquito larvae development

Another solution for improving the control of container-breeding mosquitoes in water catch basins as one of the most productive breeding sites in urban areas would be changes in their design that no water remains in the catch basin. In standard below-ground catch basins, as observed in Wrocław and in a study carried out by Kronenwetter-Koepel et al. (2005), storm water enters via a gate and a pipe system with debris and remains below the inlet/outlet pipe even during dry periods. By lowering the inlet/outlet pipe location, constant and full water flow within the underground drainage system would be provided (Fig. 4). This model prevents water and debris accumulation and thus mosquito larvae development.
